# Spectral and topological analyses of the cortical representation of the head position: Does hypnotizability matter?

**DOI:** 10.1002/brb3.1277

**Published:** 2019-04-18

**Authors:** Esther Ibáñez‐Marcelo, Lisa Campioni, Diego Manzoni, Enrica L. Santarcangelo, Giovanni Petri

**Affiliations:** ^1^ ISI Foundation Turin Italy; ^2^ Department of Translational Research and New Technologies in Medicine and Surgery University of Pisa Pisa Italy; ^3^ ISI Global Science Foundation New York NY USA

**Keywords:** EEG, head position, hypnotizability, perception, persistent homology, spectral analysis

## Abstract

**Introduction:**

The aim of this exploratory study was to assess the EEG correlates of head positions (which have never been studied in humans) in participants with different psychophysiological characteristics, as encoded by their hypnotizability scores. This choice is motivated by earlier studies suggesting different processing of vestibular/neck proprioceptive information in subjects with high *(highs)* and low *(lows)* hypnotizability scores maintaining their head rotated toward one side (RH).

**Methods:**

We analyzed EEG signals recorded in 20 *highs* and 19 *lows* in basal conditions (head forward) and during RH using spectral analysis, which captures changes localized to specific recording sites, and topological data analysis (TDA), which instead describes large‐scale differences in processing and representing sensorimotor information.

**Results:**

Spectral analysis revealed significant differences related to head position for alpha 1, beta 2, beta 3, and gamma bands, but not to hypnotizability. TDA instead revealed global hypnotizability‐related differences in the strengths of the correlations among recording sites during RH. Significant changes were observed in *lows* on the left parieto‐occipital side and in *highs* in right frontoparietal region. Significant differences between the two groups were found in the occipital region, where changes were larger in *lows* than in *highs*.

**Conclusions:**

This study reports finding of the EEG correlates of changes in the head posture for the first time, indicating that hypnotizability is related to the head posture representation/processing on large‐scale networks and that spectral and topological data analyses provide complementary results.

## 1 INTRODUCTION

The EEG correlates of the tonic vestibular/neck proprioceptive information related to head positions have never been studied in humans, despite the relevant physiological interest of this sensorimotor condition which modulates motoneuronal excitability through the activation of several brain networks (Manzoni, 2005). It has been reported, however, that the vestibular and neck proprioceptive information are conveyed to the fronto‐temporo‐parietal cortex, insula, and hippocampus (Lopez & Blanke, 2011), that galvanic vestibular stimulation induces a slight suppression of gamma power in lateral regions followed by an increase in beta and gamma power in the frontal regions, and that the power of each oscillatory band throughout frontal, central/parietal, and occipital electrodes are linearly correlated with the stimulus intensity (Kim et al., 2013).

### 1.1 Sensorimotor integration and hypnotizability

Recent evidence has shown that sensorimotor integration is modulated by the psychophysiological trait of hypnotizability (Santarcangelo & Scattina, 2016). Hypnotizability is known to predict the proneness to modify perception, memory, and behavior according to specific suggestions, and is measured by pyschometric scales. Alterations of visual and leg proprioceptive information (Santarcangelo, Scattina, Carli, Macerata, & Manzoni, 2008) and asymmetric tactile feet stimulation (Solari, Orsini, & Santarcangelo, 2016) induce larger and/or faster body sway in highly hypnotizable individuals (*highs*), while tonic neck rotation induces changes in the velocity of body sway only in low hypnotizable subjects (*lows*) (Santarcangelo et al., 2008). Hypnotizability is also associated with morphofunctional differences in the cerebral cortex (Landry, Lifshitz, & Raz, 2017) and cerebellar cortex (Picerni et al., 2018; Bocci et al., 2017), which are structures relevant to sensorimotor integration. Indeed, the vestibular and neck proprioceptive information are conveyed to the frontoparietal, insular, and cingulate cortices which show also hypnotizability‐related morphofunctional properties (Landry et al., 2017). In addition, cerebellar function, which is related to hypnotizability (Bocci et al., 2017), is required for the elaboration of sensorimotor information related to the head position (Manzoni, 2005; Kammermeier, Kleine, & Büttner, 2009). Spectral analysis of EEG signals can be used to characterize the cortical representation of the head posture. Analysis was limited to alpha and beta frequencies because they are the frequency bands most involved in any aspect of movement and posture (Enders & Nigg, 2016). In fact, beta power is reduced in all conditions related to movement actual, observed, and imagined movement/posture (Pfurtscheller, Neuper, Brunner, & Da Silva, 2005; Keinrath, Wriessnegger, Müller‐Putz, & Pfurtscheller, 2006; Turella et al., 2016), whereas it may increase during action planning without execution, which is likely due to the integration of bottom‐up sensorimotor information due to movement (Turella et al., 2016). Gamma power synchronization has been found in a wide range of cognitive (Bosman, Lansink, & Pennartz, 2014) and sensory operations (van Ede, Szebényi, & Maris, 2014). Alpha power is mainly involved in cognitive processes (Başar, Schürmann, Başar‐Eroglu, & Karakas, 1997; Klimesch, 1999) but modulation of alpha rhythms has been observed also during the elaboration of event‐specific sensory and motor information (Babiloni et al., 2016) and is influenced by the specific task and by the individual motor experience (Duru & Assem, 2018). Gamma power synchronization has been found in a wide range of cognitive (Bosman et al., 2014) and sensory operations (van Ede et al., 2014) and has been associated with imagined rather than executed motor actions (Korik, Sosnik, Siddique, & Coyle, 2018). Theta/delta modulation, however, has not been observed during movement. Hypnotizability‐related EEG spectral differences have been studied during imagery tasks (Cavallaro et al., 2010), but not during different sensorimotor conditions, with the exception of nociceptive stimulation (Zeev‐Wolf, Goldstein, Bonne, & Abramowitz, 2016) which, however, has been preferentially investigated through cortically evoked potentials (De Pascalis, Varriale, & Cacace, 2015; Valentini, Betti, Hu, & Aglioti, 2013).

### 1.2 Topological data analysis

Spectral observables characterize local changes in cortical activity, whereas topological ones, obtained from topological data analysis (TDA) techniques (See Appendix S1), are able to characterize the shape and properties of the networks active in correspondence to specific conditions on a mesoscopic scale (Sporns, 2013; Petri et al., 2014; Lord et al., 2016; Giusti, Ghrist, & Bassett, 2016; Sizemore et al., 2018), that is, by considering the different global processing schemes of vestibular/neck proprioceptive information related to the rotated position of the head (RH) in *highs* and *lows*.

Previous TDA analyses investigated global and mesoscopic properties on the active networks (Petri, Ibanez‐Marcelo, Campioni, Phingyomark, & Santarcangelo, 2018), of the cortical networks active during physically and imaginatively rotated position of the head independently from their anatomical distribution.

We take here an alternative approach by focusing on the comparison of both classical spectral analysis and the study of topological properties at the nodal level, that is, with reference to each recording site. Persistent homology (See Appendix S1), one of the main tools of TDA, describes the shape of high‐dimensional datasets by producing a series of progressively finer approximations of a given whole data space. It studies the evolution of the connectivity and lack thereof, hence holes, in all dimensions (e.g., connected components, one‐dimensional cycles, three‐dimensional cavities, and their higher dimensional analogues (Sizemore, Giusti, & Bassett, 2016) along this sequence of approximations (called a filtration). Here, the data space we focus on is the one generated by the correlation matrices between EEG signals of each subject. The persistent homology of these spaces captures the both the presences and lack of correlation patterns between multiple recording sites. Cycles, each corresponding to a region of weakened connectivity in the correlations patterns, are mesoscopic topological features which encompass multiple regions and are characterized by their appearance along the series of approximation (cycle birth, b), their disappearance (death d), the number of regions they include (cycle length), and their persistence across the filtration, defined as π = *d*−*b*, capturing how long they live. Local topological information about specific regions can be obtained from the homological scaffold (Petri et al., 2014): a network representation, where of the homological structure built by aggregating the cycles and weighting them according to their persistence. This network is composed by nodes, which represent recording sites, and edges, that represent connections between such sites. The intensity of the connection is represented by a weight assigned to the edge.

A measure of the importance of a node, which we dub nodal strength, is then obtained by summing the weights of the edges stemming from that node in the scaffold. This method has been applied to resting state fMRI data and has revealed topological correlates of altered states of consciousness (Petri et al., 2014) and epileptic seizures (Wang, Ombao, & Chung, 2015), as well as pointing to specific topological structures in resting state (Lord et al., 2016) and during attention modulation (Yoo, Kim, Ahn, & Ye, 2016).

### 1.3 Aim

The aim of this exploratory study is: on the one hand, to show from the spectral point of view the alpha, beta, and gamma frequencies correlate with the position of rotated head in *highs* and *lows*; on the other hand, we asses this difference between subjects through the topological structure extracted of the EEG signals. Moreover, we can compare the power of both techniques (spectral and topological) to characterize EEG features and distinguish between different head position and/or different groups (*highs*,* lows*).

## 2 METHODS

### 2.1 Subjects

The study was conducted in healthy unpaid volunteers according to the Declaration of Helsinki for human studies and approved by the local Ethics Committee. After signing an informed consent, hypnotizability was measured through the Italian version of the Stanford Scale of Hypnotic Susceptibility (SSHS), form A (Weitzenhoffer & Hilgard, 1959) in a sample of 200 right‐handed (Edinburgh Handedness Inventory, score (mean ± SD): 17 ± 1) students of the University of Pisa (age (mean ± SD); 22 ± 1.9 years) with negative anamnesis of medical, neurological, and psychiatric disease, not treated pharmacologically in the latest 2 weeks. No significant difference in age (females: 22 ± 1.4; males: 23 ± 0.6) and handedness (both gender: 17 ± 1) was observed between females and males. Participants were classified as high (*highs*, SHSS score ≥ 8/12), medium (mediums, SHSS score: 57), and low hypnotizable subjects (*lows*, SHSS score <4/12). From this sample, 20 consecutive *highs* (SHSS score (mean ± SD): 9.6 ± 1.4, 11 females) and 20 consecutive *lows* (SHSS, mean + SD: 1.5 ± 0.9, 11 females) were enrolled in this study. *Highs* and *lows* did not differ in age (*highs*, 21 ± 0.7; *lows* 22 ± 09) and handedness (*highs*, 17 ± 1; *lows*, 17 ± 1).

### 2.2 Experimental procedure

Experimental sessions were conducted between 11 a.m. and 2 p.m. by an experimenter not aware of the participants hypnotizability score. All participants declared the intake of one cup of coffee between 8 and 9.00 a.m., their usual caffeine intake being limited to two cups of coffee per day, no tea, and cola. During the session, participants were comfortably seated in a semi‐reclined armchair in a temperature controlled (21–22 C°), sound‐, and light‐attenuated room. After fitting the EEG montage and 5 min of familiarization with the experimental setting, they were invited to close their eyes and relax (basal conditions (B), 1 min, head forward) and then to rotate their head toward the right side to align their chin with the shoulder—maximum neck rotation allowed—and maintain this position (rotated head, RH, 1 min) till the experimenter verbal instruction (STOP) to go back to the head forward position The script of the instruction for relaxation followed the standard recommendation for simple relaxation (Benson, Arns, & Hoffman, 1981). B and RH were randomly administered. One trial was performed to avoid learning effects, which may mask possible hypnotizability‐related differences, as earlier observed for EEG (Madeo, Castellani, Santarcangelo, & Mocenni, 2013). Head position and eye closure were visually confirmed throughout the session by one of the experimenters.

### 2.3 EEG acquisition and processing

EEG was acquired at a sample rate of 1,000 Hz (Bandpass DC to 1,000 Hz) through a Quick‐CapEEG and QuickCell system (Compumedics NeuroMedical Supplies) and amplified by a Neuroscan Nunanps. EEG electrodes (*N* = 32) were placed according to the 1,020 International System (FP1, FP2, F7, F8, F3, F4, FZ, FT7, FT8, FC3, FC4, FCZ, T3, T4, C3, C4, CZ, TP7, TP8, CP3, CP4, CPZ, T5, T6, P3, P4, PZ, O1, O2, PO1, PO2, Oz). In addition, ear lobes (A1, A2), eye, and EKG electrodes (standard DI lead) were also placed. The reference electrode during acquisition was FCz; off‐line, the signal was referred to A1/A2 and FCz was restored. Filters (notch at 50 Hz, bandpass 0.5–45 Hz) were applied a‐posteriori. Electrodes impedance was kept under 10 kΩ. No participant had more than one bad channel, interpolated using the spherical interpolation method (EEGLAB pop interp function). Source components were obtained using Independent Component Analysis (infomax ICA algorithm, EEGLAB function runica). They were visually inspected to remove artifacts. The signal was divided into 20 s epochs (20'000 samples). No windowing function was applied to the raw data. The epoch selection process (deletion criteria: amplitudes ≥ ± 100 μV, median amplitude > 6SD of the remaining channels) removed a maximum of 1 epoch in each subject. The earliest less noisy 20 s interval of basal conditions and the interval comprised between the 20 and 40th sec of RH were chosen for analysis. Absolute spectral powers were estimated on two separate 10 s epochs using the Welchs method.

### 2.4 Persistent homology

In the same time, intervals topological invariants were extracted from the correlations among EEG signals during basal and task conditions. In particular, persistent homology (See Appendix S1for details) was computed as follows:

- For each subject and condition, we computed the (Pearson) correlation matrix between all pairs of EEG signals [contained in (−1,1)]. We then consider a similarity matrix defined as *S*_*ij*_ = 1−| *C*_*ij*_ |
- Each matrix was then thresholded at all distinct values between −1 and 1 to produce a sequence of approximated similarity matrices (the first matrix is empty, while the last contains the entire information). To each thresholded matrix, we can associate a new structure, called a simplicial complex, the shape and structure of which depends on the signal properties and can be characterized by the number and properties of its holes; we focus here on one‐dimensional cycles.
- Each cycle is characterized by its birth, death, length, and persistence across the sequence of thresholded matrices. For each subject, an individual homological scaffold was then constructed by aggregating all the cycles and summing the associated weights. In this way, it is possible to project the persistent homological information to the level of regions and compute the nodal strength, that is, the integrated amount of cycles that pass through each node, capturing its topological importance.
- For each region/node *r*, we constructed a vector $s_{g,c}^{r}$containing the nodal strengths of all subjects in a certain group g (*highs*,* lows*) and condition c (basal, RH). The ith entry $s_{gc,i}^{r}$ in each vector corresponded to the nodal strength of the ith subject in group g and condition *c*. We then computed node‐specific differences at the group level by measuring the Euclidean distance ($d\left(x,y\right)=\sqrt{\sum_{i}{\left(x_{i}-y_{i}\right)}^{2}}$) between the vectors corresponding to basal and task condition, where each component i corresponds to a subject in the same group. The values obtained measure the extent of the change between basal and condition at the group level for a specific region.

### 2.5 Variables and statistical analysis

The log‐transformed absolute power of beta1 (1,316 Hz), beta 2 (1,620 Hz), beta 3 (2,036 Hz), and gamma bands (36–45 Hz) recorded during simple relaxation with head forward (basal, B) and in conditions of rotated head (RH) were studied. The absolute power of each frequency band averaged across the left (Fp1, F3, F7) and right frontal (Fp2, F4, F8), left (FC3, FT7, T3) and right medioanterior (FC4, FT8, T4), left (C3,TP7, CP3) and right medioposterior (C4, TP8, CP4), left (T5, P3, PO1, O1) and right occipital (T6, P4, PO2, O2), and anterior (Fz, Fcz, Cz) and posterior midline regions (CPz, Pz, Oz) was studied. Repeated measures ANOVA (SPSS.15) according to a 2 Hypnotizability (*highs*,* lows*) × 2 Conditions (B, RH) × 2 Hemisphere (right, left) design. The Greenhouse–Geisser ε correction for non‐sphericity was applied when necessary. Post hoc comparisons were performed through paired *t* test between conditions and unpaired *t* test between groups. The same design was applied to the analysis of births, deaths, lengths, and persistences of cycles. Since the latter were mostly generated by electrodes placed in both hemispheres, the nodal strengths in the scaffolds were analyzed across a 2 Hypnotizability × 2 Condition design. For all analyses, the significance level was set at *p* = 0.05. No correction was applied owing to the exploratory nature of the study.

## 3 RESULTS

One low subject was excluded from spectral analysis due to noisy EEG signals; thus, the findings were obtained in 20 *highs* and 19 *lows*. Persistence homology could be studied in 18 *highs* and 19 *lows* because in 2 *highs* TDA did not detect cycles. The absence of detectable cycles indicate a locally uniform correlations structure (Petri et al., 2018).

### 3.1 Spectral analysis

**Figure 1:**
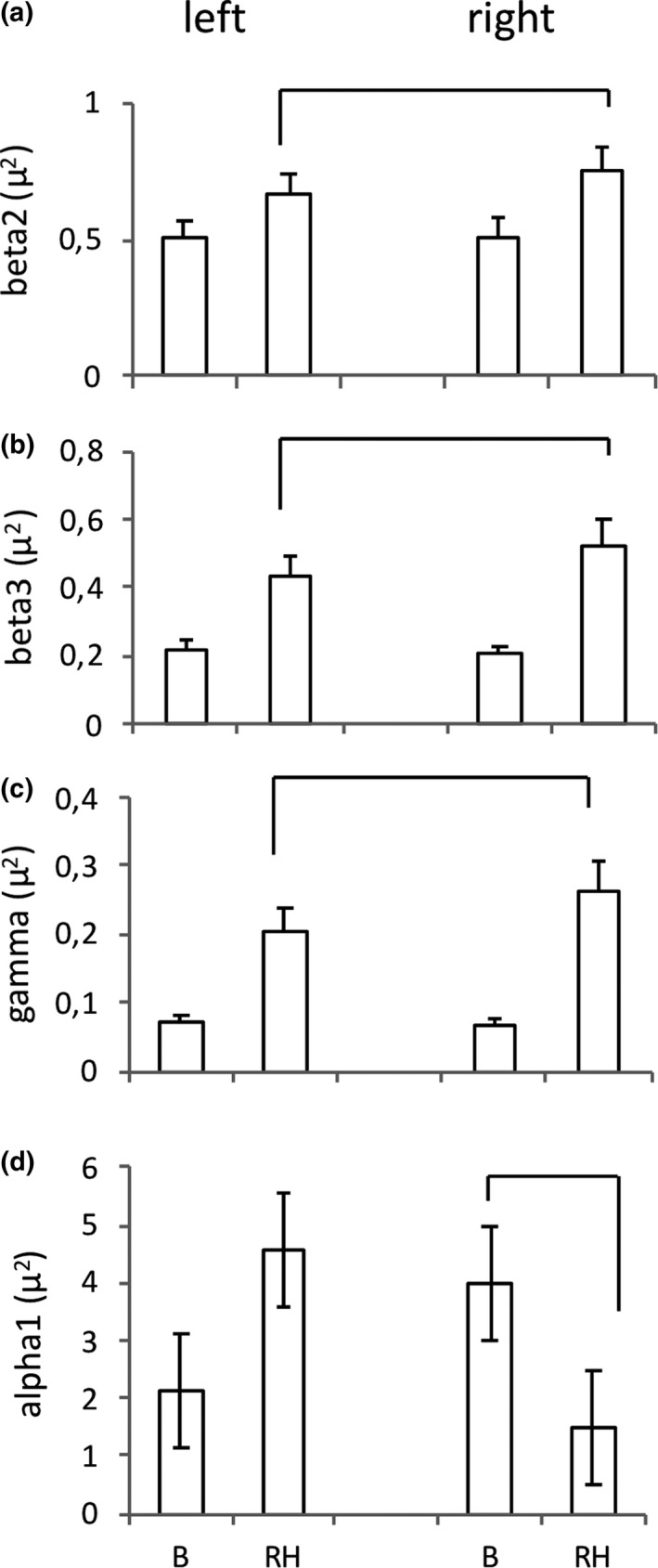
Spectral results. Original absolute power (mean, SEM), collapsed electrodes of the left and right frontal regions. (b) Basal, head forward condition; RH, rotated head. Lines indicate significant differences

Spectral analysis revealed significant differences between head positions, but not between *highs* and *lows*. As reported in Table 1, ANOVA revealed that in the frontal and medioanterior regions all frequency bands exhibited significant Conditions effects indicating that their absolute power increased during the maintenance of the rotated position of the head (RH) with respect to basal conditions (B). In the medioposterior and occipital regions, significant increases were observed for beta 2, beta 3, and gamma. Significant Condition × Hemisphere interactions (Table 2) were observed at the frontal level for beta 3 and gamma, at the medioanterior level for beta 2, beta 3, and gamma (Figure 1a–c), and at the medioposterior level for gamma. In all these regions, during RH, the EEG bands power increased on both hemispheres, but the increases were higher on the right than on the left side. In contrast, at medioposterior level a significant decrease in the alpha 1 power was observed during RH on the right hemisphere and no significant change was found on the left hemisphere (Figure 1d). On the midline, significant increases in beta 2, beta 3, and gamma power were observed during RH (Table 1).

**Table 1:** Significant condition effect

Region	Effect	Beta1	beta 2	beta 3	Gamma
Frontal	B*<*RH	*F* = 4.050, *p < *0.051, η^2^ = 0.094	*F* = 21.499, *p < *0.0001, η^2^ = 0.355	*F* = 44.68, *p < *0.0001, η^2^ = 0.539	*F* = 63.410, *p < *0.0001, η^2^ = 0.618
Medioanterior	B*<*RH	*F* = 6.145,	*F* = 30.423,	*F* = 54.514,	*F* = 69.752,
		*p < *0.018, η^2^ = 0.132	*p < *0.0001, η^2^ = 0.438	*p < *0.0001, η^2^ = 0.583	*p < *0.0001, η^2^ = 0.641
Medioposterior	B*<*RH		*F* = 27.452,	*F* = 56.135,	*F* = 81.159,
			*p < *0.0001, η^2^ = 0.413	*p < *0.0001, η^2^ = 0.590	*p < *0.0001, η^2^ = 0.675
Occipital	B*<*RH		*F* = 17.496,	*F* = 48.754,	*F* = 73.985,
			*p < *0.0001, η^2^ = 0.310	*p < *0.0001, η^2^ = 0.556	*p < *0.0001, η^2^ = 0.661
Midline	B*<*RH				*F* = 75.827,

Note

F, Fisher test. EEG bands: beta1 (13–16 Hz), beta 2 (16–20 Hz), beta 3 (20–36 Hz), gamma (36–45 Hz).

**Table 2:** Significant hemisphere × Condition interactions

Region	Effect	alpha 1	beta 2	beta 3	Gamma
Frontal	Hemi x Cond			*F* = 8.677, *p* < 0.005, η^2^ = 0.182	*F* = 5.685, *p* < 0.022, η^2^ = 0.127
				B<RH	B<RH
				RH: left< right, *t* = 2.638, *p* < 0.012	RH: left < right, *t* = 2.388, *p* < 0.022
Medioanterior	Hemi × Cond		*F* = 9.695, *p* < 0.003, η^2^ = 0.199	*F* = 18.476, *p* < 0.0001, η^2^ = 0.321	*F* = 17.389, *p* < 0.0001, η^2^ = 0.308
			B<RH	B<RH	B<RH
			RH: left <right, *t* = 2.147, *p* < 0.038	RH: left<right, *t* = 3.126, *p* < 0.003	RH: left<right, *t* = 3.514, *p* < 0.001
Medioposterior	Hemi × Cond	*F*(1, 39) = 7.631, *p* < 0.009, η^2^ = 0.164			*F* = 10.621, *p* < 0.002, η^2^ = 0.214
		Right, B>RH, *t* = 3.121, *p* < 0.003			B<RH
		RH: left < right, *t* = 1.505, *p* < 0.004			RH: left<right, *t* = 1.966, *p* < 0.056
Midline	Hemi × Cond			*F* = 4.927, *p* < 0.033, η^2^ = 0.119	
				B, anterior > posterior	
				*t* = 2.503, *p* < 0.017	

Note

Hemi, hemisphere; Cond, condition; RH, rotated head; B, baseline head forward condition; for frequencies range, see Table 1.

A significant interaction of Hypnotizability with Hemisphere was found for beta 2 at frontal level (*F* (1, 39) = 4.126, *p* < 0.049, η^2^ = 0.096). Its decomposition revealed significant lower beta 2 power on the left than on the right hemisphere in *lows* independently of the head position (*t*(1, 39) = 2.35, *p* < 0.03), whereas *highs* did not exhibit any asymmetry. Spectral analysis did not reveal any modulation of the EEG correlates of the rotated position of the head by hypnotizability.

### 3.2 Topological data analysis

Topological observables revealed significant differences between the head positions and hypnotizability groups. In both the groups of participants, the cycles were detected in the left or right hemisphere only (pure), in the left or right hemisphere together with the involvement of some central regions (non pure), and finally, that spanned regions in both left and right hemispheres (mixed). Their number, not significantly different between each other (χ2 = 1.06, *p* = 0.313), is reported in Table 3. However, the number of cycles not restricted to one hemisphere (non‐pure + mixed) was systematically larger in both groups. Thus, ANOVA was performed on the bilateral frontal, medioanterior, medioposterior, occipital, and on midline regions.

**Table 3:** Number and localization of detected cycles

Group	Condition	Pure		Non‐pure		Mixed	Non‐pure+mixed
		Left	Right	Left	Right	Left–Right	
Highs	B	9	8	12	8	20	40
	RH	10	9	19	13	17	49
Lows	B	13	10	15	11	18	44
	RH	15	10	14	14	17	45

Note

Cycles: pure, developed within one hemisphere; non‐pure, developed within one hemisphere and median structures; mixed, developed within both hemispheres; Left, Right: hemispheres; B, RH: baseline head forward, rotated head condition.

**Figure 2:**
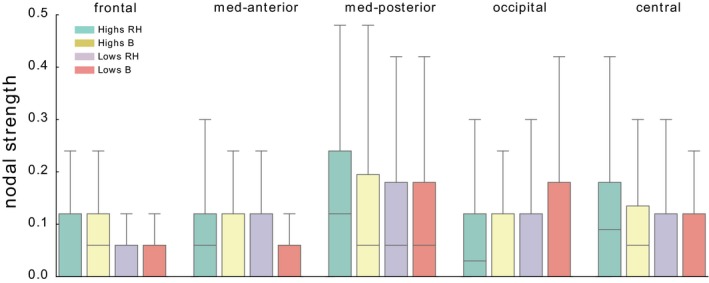
Distribution of nodal strengths. We show the distributions of nodal strengths in the scaffolds associated to the four conditions *highs/lows* B/RH. Nodes are grouped as follows. Frontal FP1, FP2, F8, F7, F4, F3; medioanterior: T3, FT7, T4, FC4, FT8, FC3; medioposterior: C4, C3, CP3, CP4, TP8, TP7; occipital: O1, P4, P3, PO2, O2, T6, PO1, T5; central: CZ, PZ, FZ, OZ, CPZ, FCZ

Significant differences in the nodal strength between RH and B were observed in *highs* on right frontoparietal sites (F8, B>RH, *t* = −3.57, *p* = 0.002); P4, B<RH (*t* = 2.4726, *p* = 0.024) and in *lows* on the left parietooccipital sites (T5, B>RH, *t* = 2.480, *p* = 0.023; PO1, B>RH, *t* = −2.453, *p* = 0.024), respectively (Figure 2).

**Figure 3:**
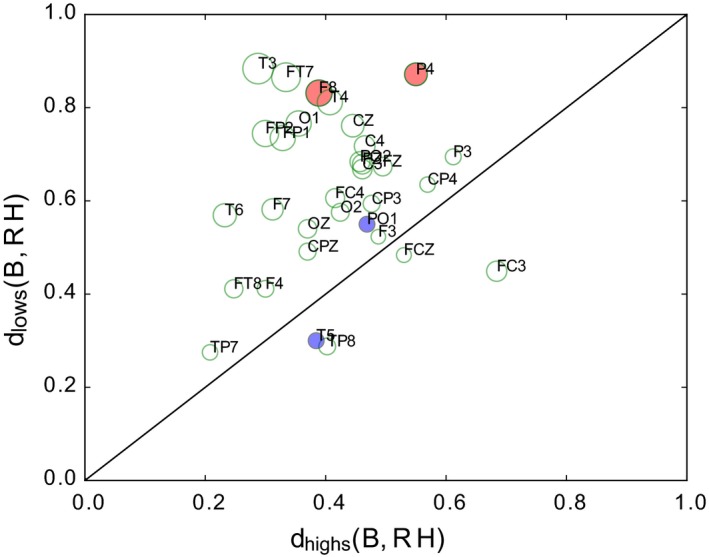
Difference between Euclidean distance of nodal strength vectors. The size of the dots is proportional to their distance from the diagonal, thus smaller points indicate smaller differences between B–RH distances. Most of the points remain in the upper diagonal part, which indicates smaller differences between RH and B in *highs*. Red and blue circles indicate significant nodal strength differences between RH and B in both groups

Furthermore, as shown by the Euclidean distances computed between the vectors of nodal strength between RH and B, we found that the changes occurring in *highs* were systematically smaller than those occurring in *lows* (Figure 3).

Collapsing nodes by regions for each hemisphere (Figure 3), a significant Hypnotizability x Condition interaction was observed at the occipital level (*F*(1, 37) = 2.683, *p* = 0.046). Its decomposition revealed only a significant difference between *highs* and *lows* during RH (*t* = 2.67, *p* = 0.008) in the presence of similar values in basal conditions.

## 4 DISCUSSION

The study provides the first report on the cortical representation of the sensorimotor information associated with the rotated position of the head. Spectral analysis captured only differences between head positions while persistence homology revealed also hypnotizability‐related differences. Spectral analysis showed that the EEG power of high frequency bands increased during the maintenance of the rotated position of the head. This agrees with the beta synchronization observed during focused motor attention (Kristeva‐Feige, Fritsch, Timmer, & Lücking, 2002) and in the maintenance of postures (Gilbertson et al., 2005; Schoffelen, Oostenveld, & Fries, 2005), which may be accounted for by earlier observations of synchronous firing of neurons during sustained contractions (Conway et al., 1995) and slow movements of hand muscles (Salenius, Portin, Kajola, Salmelin, & Hari, 1997). In contrast, alpha power decreased in the medioposterior region, in line with the hypothesized inhibitory function of the alpha rhythm (Palva & Palva, 2007, 2011). Since the increases in beta and gamma power were larger on the right side (frontal and medioanterior regions) and the alpha power decreases were found only on this side (medioposterior region), we argue that the observed EEG changes do represent the sensorimotor information associated with the rotated head posture. The asymmetric changes in the EEG power could be due to the larger proprioceptive information arising from the lengthened left neck muscles and are in line with the gamma power increases observed contralaterally to a sustained tactile stimulation (van Ede et al., 2014). Nonetheless, in the high frequency bands, the rotated head position was characterized by power increases on both sides, although larger on the right one. This may be accounted for by the two different processes characterizing our task. One was cognitive and consisted of directing attention to maintain the head rotated toward one side, and the other was sensorimotor and consisted of the cortical representation of the sensorimotor asset relative to the head position. In fact, in the frontal regions, lower beta (beta1 and beta 2) and gamma did not exhibit any asymmetry associated with the rotated position of the head, which is consistent with the involvement of the anterior brain region in cognitive rather than sensory processes and with the bilateral representation of the cognitive processes related to movement and posture (Cremades & Pease, 2007). Interestingly, a bilateral representation of the rotated position of the head was observed also at the occipital level, where beta 2, beta 3, and gamma power were influenced by the head position despite the major visual competence of this brain region. A contribution of the occipital region, may be due to cross‐modal sensory activation (Heimler, Striem‐Amit, & Amedi, 2015) which has been observed in several experimental protocols such as sighted adults who recruit the ventral visual cortex during tactile Braille reading (Bola et al., 2016) and, for the auditory modality, congenitally deaf subjects showing activation of the auditory cortex during tactile stimulation (Levänen, Jousmäki, & Hari, 1998; Poirier et al., 2005). Nonetheless, since the occipital increases in beta and gamma were bilateral and symmetric, we hypothesize that they could be due also to a supramodal representation of the sensory and imaginative context, independent from the specific sensory modality (Bonino et al., 2015; Papale, Chiesi, Rampinini, Pietrini, & Ricciardi, 2016).

The other process that is the sensory–motor representation of the rotated head could have its correlates in the asymmetric increases in beta 2, beta 3, and gamma power in the medioanterior region (Turella et al., 2016; van Ede et al., 2014). Finally, the power increases observed on the midline sites can be related to both cognitive and sensory aspects of the task (Başar et al., 1997; Klimesch, 1999). The scarce hypnotizability‐related differences observed are in line with earlier studies of cognitive tasks characterized by more widespread changes within *highs* than within *lows* and the absence of local significant differences between *highs* and *lows* (Cavallaro et al., 2010). With respect to spectral analysis, persistent homology provides a different perspective of the EEG changes occurring in the present study because it reveals the strength of the relation between cortical sites and can suggest the mechanisms leading to the observed spectral changes. It is more sensitive than spectral analysis to the hypnotizability‐related changes in the sensorimotor information associated with the position of rotated head. The Euclidean differences between the nodal strength in RH with respect to basal conditions (Figure 3), in fact, were almost always lower in *highs* than in *lows*. Thus, on one hand, the present study supports earlier findings showing larger changes in *lows* than in *highs* during both the real and imagined rotated position of the head (Petri et al., 2018). On the other hand, it reveals spatial differences and different changes between the two groups, as *highs* decreased their nodal strength at right frontoparietal sites and *lows* at left parieto‐occipital sites. In line with this observation, collapsing sites of each region a significant difference between groups during head rotation was found in the occipital region, which suggests that the maintenance of the rotated posture of the head was associated in *lows* with its visual representation and in *highs* with a preferential kinaesthetic representation, and is in line with the preferential sensory modality of imagery reported by *highs* and *lows* in earlier experiments (Santarcangelo et al., 2010) and with recent findings reporting greater effects of kinesthetic imagery on corticospinal excitability in *highs* (Cirillo, Srzich, Byblow, Stinear, & Anson, 2018).

## 5 LIMITATIONS AND CONCLUSIONS

A limitation of the study is the absence of medium hypnotizable participants. According to the many reports of Gaussian distribution of hypnotizability (De Pascalis, Bellusci, & Russo, 2000; Carvalho, Kirsch, Mazzoni, & Leal, 2008), they could better represent the general population. Nonetheless, bimodal distribution of hypnotizability showing a larger percentage of low‐to medium hypnotizable individuals, have also been reported (Balthazard & Woody, 1989). Thus, we think that our findings in *lows* are reliably referable to the general population. Another limitation is the low number of recording sites with the absence of source analysis and of electromyographic recording of neck muscles and of eye movements monitoring. EMG would allow to test the hypothesis of greater coherence between EEG beta and muscle activity which has been observed during the maintenance of postures (Conway et al., 1995). Despite these limitations, this is the first study providing findings concerning the EEG representation of the integrated static vestibular/neck proprioceptive information. In addition, it indicates hypnotizability‐related differences in line with earlier observations of hypnotizability‐related differences in the sensorimotor domain (Santarcangelo & Scattina, 2016; Petri et al., 2018). Finally, it shows that TDA is more powerful than spectral analysis in capturing such differences. In conclusion, findings support the view that hypnotizability is associated with physiological characteristics apparently unrelated to the proneness to accept suggestions (Santarcangelo & Scattina, 2016), and thus, it may be relevant to additional aspects of everyday life.

## CONFLICTS OF INTEREST

The authors declare no conflicts.

## AUTHOR CONTRIBUTION

ELS designed the experiment; EIM and GP designed the analytical framework; LC and ELS conducted the experiment and preprocessed the data; EIM and GP analyzed the results. All authors contributed to writing the paper and approved the final manuscript.
