# Supplementary material for: Spectral and topological analyses of the cortical representation of the head position: Does hypnotizability matter?
Source: Brain Behav. 2019 Apr 18;9(6):e01277. doi: 10.1002/brb3.1277 (PMC6576149; doi:10.1002/brb3.1277)
Supplement: Supplementary file 1 [file BRB3-9-e01277-s001.pdf]

Appendix A

Mathematical formulation of homology and persistent homology

We provide here a few elementary notions required for the paper, a thorough review can be found in [1] and [2]. We will limit ourselves to the following notions:

- A *clique* is a subset of vertices such that they induce a complete subgraph (K_i is the full connected graph of i nodes). That is, every two distinct nodes in the clique are adjacent.
- A *k-simplex* is a set of $k+1$ vertices $\sigma = [p_0, \dots, p_{k-1}]$. It is easy to see that it is possible to map k-cliques onto $(k-1)$-simplices.
- A *simplicial complex* is a topological space constructed by simplices where simplices are points, lines, triangles, and their n-dimensional counterparts. We obtain a simplicial complex from a binary network by mapping k-cliques to $(k-1)$-simplices.
- A *filtration* used for the computation of persistent homology consists in a family of ordered clique complexes, X_i one inside the other ($\cdots \subseteq X_{i-1} \subseteq X_i \subseteq X_{i+1} \subseteq \dots$) obtained from the progressive thresholding of a weighted network [3].

We then defined the set of n-dimensional chains $C_n(X)$ of a simplicial complex X as the formal sums of n-simplices:

$$C_n(X) = \{r_1\sigma_1 + r_2\sigma_2 + ... | r_i \in \mathbb{Z}, \sigma_i \in X_n\}. \tag{1}$$

The *boundary map* ∂_n between n-dimensional chains $C_n(X)$ to $(n-1)$-dimensional chains $C_{n-1}(X)$ corresponds to the intuitive notion of boundary of a shape:.

$$\partial_n : \quad C_n(X) \to C_{n-1}(X) \tag{2}$$

$$[v_1, \dots, v_n] \mapsto \sum_{i=0}^{n} (-1)^i [v_0, \dots, \hat{v}_i, \dots, v_n] \tag{3}$$

where the hat denotes the omission of the vertex. It is easy to see that $\partial_n \partial_{n+1} = 0 \quad \forall n$, that is a boundary has no boundary.

A simplicial complex X induces the *chain complex*, $\cdots \to C_{n+1} \to C_n \to C_{n-1} \to \cdots$ through boundary maps $...\partial_{n+2}, \partial_{n+1}, \partial_n, \partial_{n-1}, ...$

The *n-homology* of this complex is defined as the quotient of two vector spaces: the kernel of the map ∂_n quotiented by the image of the boundary map one upper dimension, ∂_{n+1},

$$H_n(X) = \ker \partial_n / \text{ im } \partial_{n+1}, \tag{4}$$

where n indicates the dimension of the generators in the homology group. We call the kernel $\ker \partial_n$ the nth cycle module as it usually denoted by Z_n, while the image im ∂_n is the nth boundary module, denoted by B_n.

For a simplicial complex X, a filtration is a totally ordered set of subcomplexes $X_i \subset X$, that starts with the empty complex and ends with the complete complex, indexed by the nonnegative integers, such that:

$$\emptyset = X_0 \subseteq X_1 \subseteq ... \subseteq X_m = X \tag{5}$$

such that if $i \leq j$ then $X_i \subseteq X_j$.

In order to define *persistent homology* [1, 4], we use superscripts to denote the index in a filtration. The ith simplicial complex X_i in a filtration gives rise to its own chain complex $(C^i_\bullet, \partial^i_\bullet)$ and the kth chain, cycle, boundary and homology modules are denoted by C^i_k, Z^i_k, B^i_k and H^i_k, respectively.

For a positive integer p, *the p-persistent kth homology* module of X_i is

$$H^{i,p}_k = Z^i_k \;/(B^{i+p}_k \cap Z^i_k). \tag{6}$$

The expression for $H^{i,p}_k$ is reminding of the expression for H^i_k, with the notable difference that it characterizes the k-cycles in the X_i subcomplex that are not the boundary of any $(k+1)$-chain from the larger complex X_{i+p}, rather than those not coming from a $(k+1)$-chain in X_i. In this way $H^{i,p}_k$ characterizes the k-dimensional holes in X_{i+p} that persisted from the subcomplex X_i.

The output of persistent homology can be summarized in a topological invariant called persistence diagram. A *persistence diagram* is a set of tuples (b, d), that describe the appearance b (birth step) and disappearance d (death step) of each hole along the filtration, with holes having longer *persistence* $\pi = d - b$ being generally considered more relevant.

A second homological summary obtained from persistent homology is the *persistence homological scaffold*, defined for the first homology group and introduced by Petri et al. [3]. It is usually used when the original data come in the form of a network and allows to re-encode some of the information from persistent homology in a more easily interpretable network format.

It is built as follows: we consider only the case of H_1; for each hole c we assign the shortest representative cycle and weigh its edges according to the holes' persistence π_c.

Given a graph G, *persistence homological scaffold* $\mathcal{H}^p_G$, is the network composed of all the cycle c corresponding to generators in H_1 weighted by their persistence. When an edge e belongs to multiple cycles $c_0, c_1, \ldots, c_s$ its weight is defined as the sum of the generators persistence:

$$\omega^\pi_e = \sum_{c_i | e \in c_i} \pi_{c_i} \tag{7}$$

Given a weighted network G, with a set of nodes n_i the *nodal strength*, ns_i, is the sum of weights of all incoming edges to n_i:

$$ns_i = \sum_{j, j \to i} w_{ij} \tag{8}$$

References

[1] Herbert Edelsbrunner and John Harer. Persistent homology-a survey. *Contemporary mathematics*, 453:257–282, 2008.

[2] Allen Hatcher. *Algebraic topology.* Cambridge University Press, 2002.

[3] Giovanni Petri, Paul Expert, Federico Turkheimer, Robin Carhart-Harris, David Nutt, Peter J Hellyer, and Francesco Vaccarino. Homological scaffolds of brain functional networks. *Journal of The Royal Society Interface*, 11(101):20140873, 2014.

[4] Afra Zomorodian and Gunnar Carlsson. Computing persistent homology. *Discrete & Computational Geometry*, 33(2):249–274, 2005.
